# Association between the non-HDL-cholesterol-to-HDL-cholesterol ratio and the risk of gallbladder polyp formation among men: a retrospective cohort study

**DOI:** 10.1186/s12944-020-01322-7

**Published:** 2020-06-23

**Authors:** Xiaofang Zhao, Huabo Zheng, Shengshuai Shan, Kun Wang, Meng Zhang, Songpu Xie, Chengyun Liu

**Affiliations:** 1 Department of Geriatrics, Union Hospital, Tongji Medical College, Huazhong University of Science and Technology, Wuhan, 430022 China; 2 The First People’s Hospital of Jiangxia District, Wuhan City & Union Jiangnan Hospital, HUST, Wuhan, 430200 China

**Keywords:** Gallbladder polyps, Non-HDL-c/HDL-c ratio, Cholesterol, Middle aged, Chinese men, Risk factors

## Abstract

**Background:**

Dyslipidaemia and male sex are associated with gallbladder polyp (GBP) formation. However, the potential relation between the non-high-density lipoprotein-cholesterol-to-high-density lipoprotein-cholesterol (non-HDL-c/HDL-c) ratio and GBPs in men is unclear.

**Methods:**

A total of 1866 eligible subjects were selected for this retrospective cohort study from Wuhan Union Hospital between April 1, 2013, and November 30, 2014. Clinical and laboratory data of subjects were collected. Patients with GBPs or cholecystectomy at baseline, with missing data for baseline lipid profiles, following abdominal ultrasonography or taking lipid-lowering drugs were excluded. The patients were divided into five groups based on their non-HDL-c/HDL-c ratios, and descriptive analyses of the baseline data were performed. A Cox proportional hazards model was applied to estimate the relationship between the non-HDL-c/HDL-c ratio and GBPs.

**Results:**

After a median follow-up of 1 year, 7.34% (*n* = 137) of the subjects developed GBPs. Compared with subjects without GBPs, those who developed GBPs after follow-up had significantly higher triglyceride (TG) levels and non-HDL-c/HDL-c ratios. The prevalence of GBPs showed a linearity increment with age, peaked in the 30–39 years group, 40–49 years group and 50–59 years group, and then declined slightly. The results of univariate analysis showed that the non-HDL-c/HDL-c ratio (hazard ratio (HR) = 1.29, 95% confidence interval (CI), 1.05–1.60, *P* = 0.0159) was positively correlated with GBPs. In the fully adjusted Cox regression model, the HRs were 2.24 for quintile 2 (95% CI: 1.13–4.44, *P* = 0.0203), 1.50 for quintile 3 (95% CI: 0.73–3.10, *P* = 0.269), 2.52 for quintile 4 (95% CI: 1.26–5.01, *P* = 0.0087) and 2.13 for quintile 5 (95% CI: 1.04–4.37, *P* = 0.0397). No interaction was found among the subgroups.

**Conclusions:**

A higher non-HDL-c/HDL-c ratio is independently related to a higher risk of GBP formation in Chinese men. Further research is needed to investigate whether this association exists in different regions and races.

## Background

GBPs are elevations of the gallbladder mucosa that protrude into the gallbladder lumen. The estimated prevalence of GBPs in the world is approximately 5% [1]. The incidence of GBPs is approximately 3–7% in abdominal ultrasound scans and 2–12% in cholecystectomy specimens [2]. The incidence of GBPs detected by abdominal ultrasound varies with the study population. The prevalence of GBPs ranges from 1.0–6.9% in the West [3]. GBPs have been reported to have prevalences of 1.4 and 6.1% in Germany [4, 5]. Zheng et al. [6] reported that the incidence of GBPs was 7.3% in a large Chinese population.

A European guideline for the management and follow-up of GBPs reported that cholecystectomy was advised if the polyp was larger than 10 mm or the polyp was larger than 6 mm with risk factors, and all other polyps were recommended for monitoring with 5 years of follow-up [7]. Unlike gallstones, GBPs are often ignored on account of lacking significant clinical signs or symptoms. GBPs have malignant potential. Gallbladder cancer progresses rapidly and has a poor prognosis, with a five-year survival rate less than 5% [8, 9]. Thus, it is necessary to identify the possible risk factors related to GBPs. Previous studies have suggested some factors related to GBPs, including male sex, middle age, hepatitis B surface antigen (HBsAg) positivity, cholecystitis, and glucose intolerance [3, 10–21].

Non-high-density lipoprotein-cholesterol (non-HDL-c), which refers to total cholesterol (TC) minus high-density lipoprotein-cholesterol (HDL-c), includes cholesterol in atherogenic lipoproteins. Evidence indicates that compared with traditional cholesterol parameters, the non-HDL-c/HDL-c ratio is a superior marker for coronary heart disease, chronic kidney disease, metabolic syndrome, and insulin resistance [22–25]. However, many studies in China have reported that male sex, low HDL-c level and dyslipidaemia were associated with a high risk of GBP formation [3, 6, 10–13]. To date, the relationship between the non-HDL-c/HDL-c ratio and GBPs among men is still unknown. Accordingly, this retrospective study aimed to evaluate the correlation between the non-HDL-c/HDL-c ratio and the risk of GBP formation among men in China.

## Methods

### Subjects

The participants were residents aged 22 to 93 who were included from the physical examination centre of Wuhan Union Hospital between April 1, 2013, and November 30, 2014. Men and subjects undergoing at least one follow-up assessment entered the final analysis. Subjects meeting the following criteria were excluded: (1) those with GBPs or cholecystectomy at baseline; (2) those with missing data for baseline lipid profiles or following abdominal ultrasonography; and (3) those taking lipid-lowering drugs. Finally, a total of 1866 subjects were eligible to participate. The Ethics Committee of Wuhan Union Hospital approved the study protocol. Written informed consent was waived due to the anonymity of the data [26].

### Physical examination and laboratory assessments

Clinical examinations were performed by trained research practitioners in the morning after an overnight fast. The examination was composed of a blood draw, anthropometry, and a health habit inventory. Anthropometric examinations included weight and height. Body mass index (BMI) was calculated based on height and weight. Fasting blood samples were used for the analysis of biochemical values by standard laboratory procedures (Beckman Coulter chemistry analyser AU5800 series, Tokyo, Japan). The parameters included TC, low-density lipoprotein-cholesterol (LDL-c), HDL-c, TG, aspartate aminotransferase (AST), alanine aminotransferase (ALT), alkaline phosphatase (ALP), gamma-glutamyl transpeptidase (GGT), uric acid (UA), and fasting plasma glucose (FPG). HBsAg was measured by an enzyme-linked immunosorbent assay. A questionnaire was given to all the subjects. The questionnaire included several questions, such as history of hypertension, lipid-lowering drug use, history of cholecystectomy, and smoking and drinking habits. Smoking and drinking were categorized into two groups: nonsmoker (never, seldom) vs smoker (sometimes, often, always) and nondrinker (never, seldom) vs drinker (sometimes, often, always). The reference value ranges were applied according to the standards of the Laboratory Department of Wuhan Union Hospital. All the observed variables were tested at the initial ultrasound time-point.

### Diagnostic criteria and definitions

GBPs and thickening of the gallbladder wall (TGW) were diagnosed by abdominal ultrasonography (MINDRAY, DC-8, China). GBPs were defined based on the following criteria: hyperechoic immobile echoes protruding from the gallbladder wall into the lumen; immobile echoes; and no acoustic shadow. TGW was diagnosed as a wall thickness of > 3 mm. The minimal time interval of ultrasound follow-up was at least 6 months. TGW was determined based on initial abdominal ultrasonography. GBP presence was determined based on the final abdominal ultrasonography.

### Statistical analysis

**Fig. 1:**
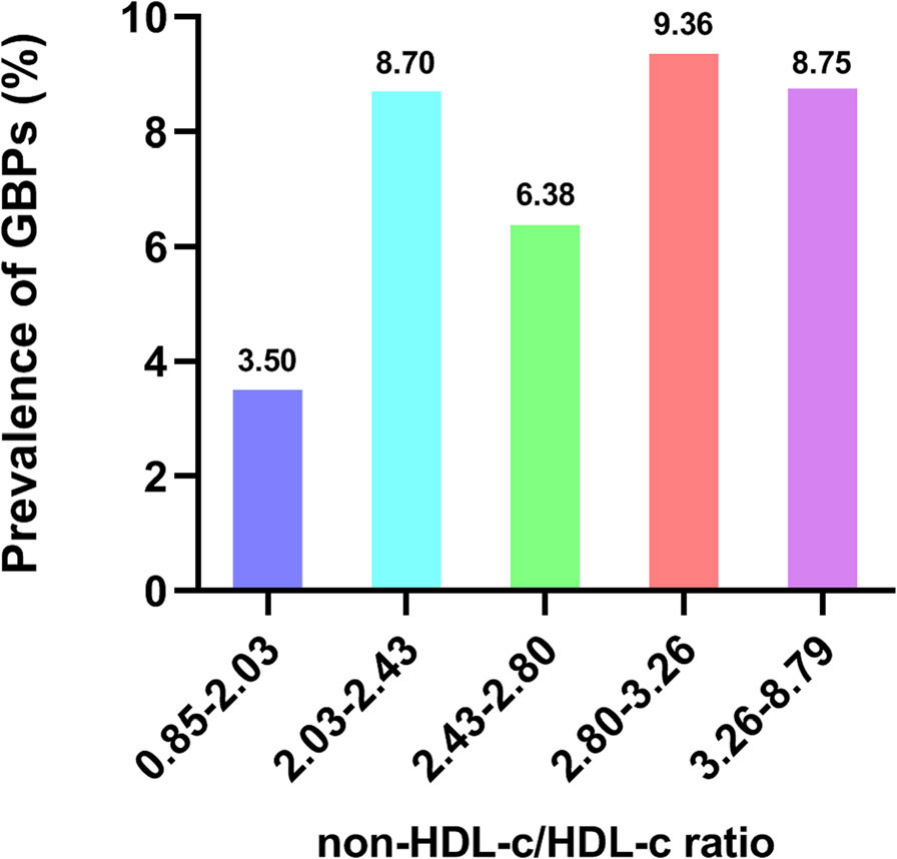
The prevalence of GBPs according to the non-HDL-c/HDL-c ratio quintiles. The number above each bar graph represents the prevalence of GBPs according to the non-HDL-c/HDL-c ratio quintiles

**Fig. 2:**
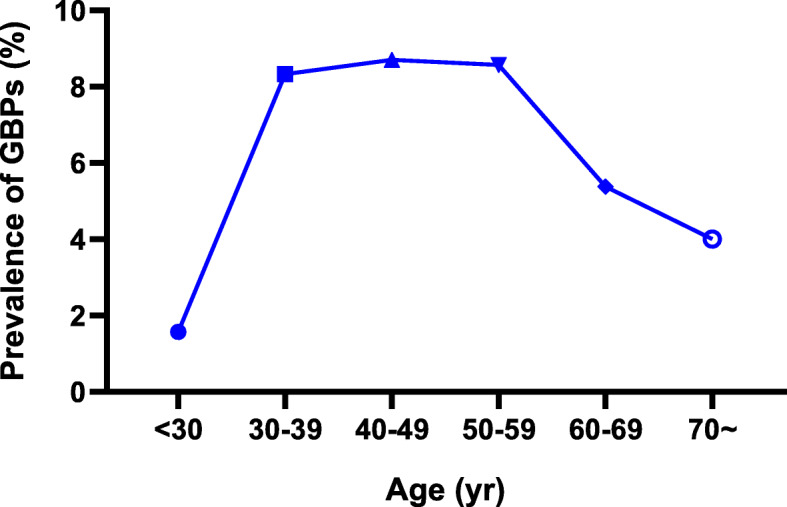
The prevalence of GBPs according to age groups

**Fig. 3:**
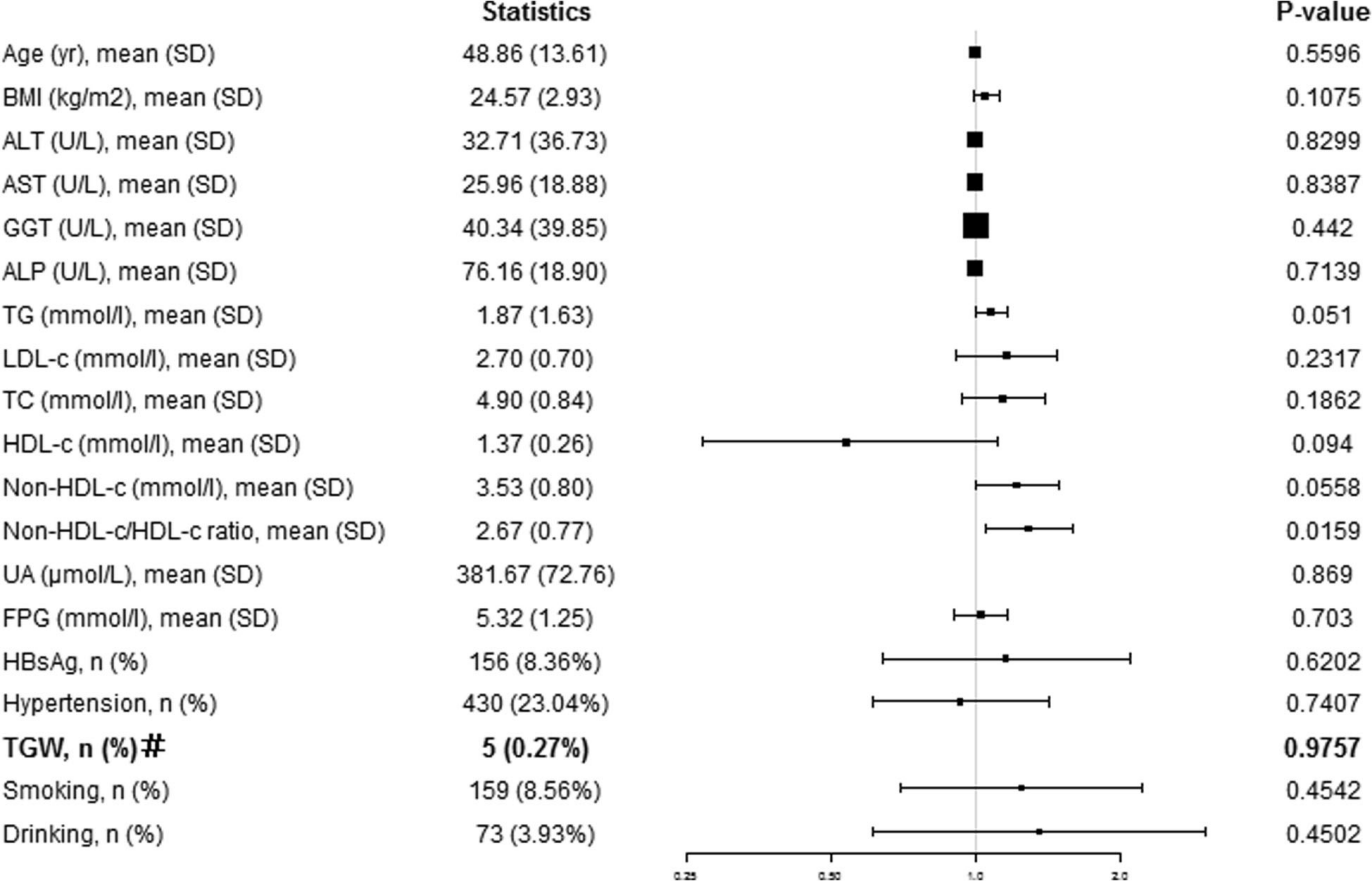
The unadjusted association between baseline variables and GBPs at the end of follow-up. Data expressed as mean (SD), or percentage. GBPs, gallbladder polyps; BMI, body mass index; ALT, alanine aminotransferase; AST, aspartate aminotransferase; GGT, gamma-glutamyl transpeptidase; ALP, alkaline phosphatase; TG, triglyceride; LDL-c, low-density lipoprotein-cholesterol; TC, total cholesterol; HDL-c, high-density lipoprotein -cholesterol; UA, uric acid; FPG, fasting plasma glucose; HBsAg, hepatitis B surface antigen; TGW, thickening of the gallbladder wall. # The model failed because of the small sample size

The total procedure of statistical analysis included seven steps. First, the mean (continuous variables) or percentage (categorical variables) was used to describe baseline characteristics and GBP status at the end of follow-up stratified by the non-HDL-c/HDL-c quintiles (Table 1). One-way ANOVA for normally distributed continuous variables, Kruskal-Wallis test for skewed continuous variables, and Chi-squared test for categorical variables were used to analyse significant differences between groups. Second, Fig. 1 illustrates the prevalence of GBPs according to non-HDL-c/HDL-c ratio quintiles. Third, the characteristics of participants with or without GBPs after follow-up were compared (Table 2). Fourth, Fig. 2 describes the prevalence of GBPs according to age group. Fifth, a univariate analysis model was applied to determine the significance of the relation between the non-HDL-c/HDL-c ratio and GBPs as well as the other independent variables (Fig. 3). Sixth, Cox proportional hazards models were applied to verify the relationship of the non-HDL-c/HDL-c ratio as a continuous variable and as categorized into quintiles with incident GBPs (Table 3). The results of unadjusted, minimally adjusted analyses and fully adjusted analyses are simultaneously shown based on the STROBE statement. The minimally adjusted regression model consisted of age and BMI. In the fully adjusted regression model, age, BMI, ALT, AST, GGT, ALP, TG, UA, FPG, HBsAg, hypertension, TGW, smoking and drinking were included. The lowest quintile was the reference for the non-HDL-c/HDL-c ratio. Seventh, the subgroup analyses were performed using Cox proportional hazards models stratified by age, BMI, HBsAg, hypertension, TGW, smoking and drinking (Table 4). All *P* values less than 0.05 (two-sided) were considered statistically significant. All statistical analyses were performed with Empower (R) (www.empowerstats.com, X & Y solutions, Inc., Boston MA) and R software (http://www.R-project.org).

**Table 1:** Baseline characteristics and GBP status at the end of follow-up by quintiles of non-HDL-c/HDL-c ratio

Variables	Non-HDL-c/HDL-c ratio					*P*-value
	Quintile 1	Quintile 2	Quintile 3	Quintile 4	Quintile 5	
Non-HDL-c/HDL-c ratio	0.85–2.03	2.03–2.43	2.43–2.80	2.80–3.26	3.26–8.79	
Sample size	371	368	376	374	377	
Age (yr), mean (SD)	50.47 (15.23)	48.06 (13.60)	49.02 (13.26)	49.01 (13.27)	47.74 (12.48)	0.059
BMI (kg/m^2^), mean (SD)	23.21 (3.04)	24.13 (2.75)	24.73 (2.82)	25.21 (2.84)	25.54 (2.63)	< 0.001
ALT (U/L), mean (SD)	23.77 (13.60)	29.91 (18.33)	36.30 (66.72)	34.67 (26.17)	38.75 (30.34)	< 0.001
AST (U/L), mean (SD)	22.97 (7.49)	25.50 (12.87)	26.72 (20.13)	26.28 (11.57)	28.28 (31.71)	< 0.001
GGT (U/L), mean (SD)	29.91 (22.58)	39.66 (44.79)	42.53 (46.60)	45.48 (42.46)	43.97 (36.17)	< 0.001
ALP (U/L), mean (SD)	76.13 (18.69)	75.01 (18.20)	75.96 (19.65)	77.02 (18.69)	76.66 (19.26)	0.655
TG (mmol/l), mean (SD)	1.08 (0.58)	1.50 (0.89)	1.79 (1.10)	2.08 (1.20)	2.87 (2.76)	< 0.001
LDL-c (mmol/l), mean (SD)	2.15 (0.49)	2.49 (0.52)	2.72 (0.56)	2.96 (0.58)	3.17 (0.79)	< 0.001
TC (mmol/l), mean (SD)	4.34 (0.64)	4.63 (0.68)	4.85 (0.66)	5.12 (0.69)	5.55 (0.92)	< 0.001
HDL-c (mmol/l), mean (SD)	1.63 (0.27)	1.43 (0.21)	1.35 (0.18)	1.28 (0.17)	1.17 (0.17)	< 0.001
Non-HDL-c (mmol/l), mean (SD)	2.71 (0.47)	3.19 (0.48)	3.50 (0.48)	3.84 (0.52)	4.38 (0.80)	< 0.001
UA (μmol/L), mean (SD)	363.45 (65.84)	373.55 (74.59)	380.48 (73.29)	391.24 (70.08)	399.19 (74.44)	< 0.001
FPG (mmol/l), mean (SD)	5.13 (0.96)	5.22 (1.05)	5.33 (1.19)	5.38 (1.50)	5.55 (1.45)	< 0.001
HBsAg, n (%)	42 (11.32)	29 (7.88)	32 (8.51)	28 (7.49)	25 (6.63)	0.184
Hypertension, n (%)	89 (23.99)	67 (18.21)	88 (23.40)	95 (25.40)	91 (24.14)	0.165
TGW, n (%)	0 (0.00)	1 (0.27)	0 (0.00)	2 (0.53)	2 (0.53)	0.409
Smoking, n (%)	27 (7.30)	27 (7.36)	29 (7.75)	35 (9.43)	41 (10.93)	0.308
Drinking, n (%)	12 (3.24)	18 (4.90)	16 (4.28)	17 (4.58)	10 (2.67)	0.477
GBPs, n (%)	13 (3.50)	32 (8.70)	24 (6.38)	35 (9.36)	33 (8.75)	0.012

Data are expressed as the mean (SD), median (interquartile range), or percentage

*BMI* body mass index, *ALT* alanine aminotransferase, *AST* aspartate aminotransferase, *GGT* gamma-glutamyl transpeptidase, *ALP* alkaline phosphatase, *TG* triglyceride, *LDL-c* low-density lipoprotein-cholesterol, *TC* total cholesterol, *HDL-c* high-density lipoprotein –cholesterol, *UA* uric acid, *FPG* fasting plasma glucose, *HBsAg* hepatitis B surface antigen, *TGW* thickening of the gallbladder wall, *GBPs* gallbladder polyps

**Table 2:** Baseline variables according to with or without GBPs after follow-up

Variable	Without GBPs after fellow-up (*n* = 1729)	With GBPs after follow-up (*n* = 137)	*P*-value
Age (yr)	48.91 ± 13.79	48.20 ± 11.13	0.56
BMI (kg/m^2^)	24.54 ± 2.95	24.98 ± 2.70	0.107
ALT (U/L)	32.77 ± 37.73	32.07 ± 20.34	0.319
AST (U/L)	25.98 ± 18.96	25.64 ± 17.92	0.33
GGT (U/L)	40.14 ± 39.12	42.86 ± 48.20	0.178
ALP (U/L)	76.12 ± 18.97	76.73 ± 18.11	0.714
TG (mmol/l)	1.85 ± 1.58	2.14 ± 2.10	0.028
LDL-c (mmol/l)	2.70 ± 0.69	2.77 ± 0.74	0.232
TC (mmol/l)	4.89 ± 0.83	4.99 ± 0.86	0.186
HDL-c (mmol/l)	1.37 ± 0.26	1.33 ± 0.25	0.094
Non-HDL-c (mmol/l)	3.52 ± 0.80	3.66 ± 0.81	0.056
Non-HDL-c/HDL-c ratio	2.65 ± 0.77	2.82 ± 0.76	0.016
UA (μmol/L)	381.59 ± 73.19	382.65 ± 67.36	0.869
FPG (mmol/l)	5.32 ± 1.24	5.36 ± 1.45	0.703
HBsAg, n (%)	143 (8.27%)	13 (9.49%)	0.62
Hypertension, n (%)	400 (23.13%)	30 (21.90%)	0.741
TGW, n (%)	5 (0.29%)	0 (0.00%)	0.529
Smoking, n (%)	145 (8.43%)	14 (10.29%)	0.453
Drinking, n (%)	66 (3.83%)	7 (5.15%)	0.448

Data expressed as mean ± SD or percentage

*GBPs* gallbladder polyps; BMI, body mass index, *ALT* alanine aminotransferase, *AST* aspartate aminotransferase, *GGT* gamma-glutamyl transpeptidase, *ALP* alkaline phosphatase, *TG* triglyceride, *LDL-c* low-density lipoprotein-cholesterol, *TC* total cholesterol, *HDL-c* high-density lipoprotein –cholesterol, *UA* uric acid, *FPG* fasting plasma glucose, *HBsAg* hepatitis B surface antigen, *TGW* thickening of the gallbladder wall

**Table 3:** Risk association between baseline non-HDL-c/HDL-c ratio with GBPs

	Unadjusted	*P*-value	Model 1^a^	*P*-value	Model 2^b^	*P*-value
	Hazard ratio (95% CI)		Hazard ratio (95% CI)		Hazard ratio (95% CI)	
Non-HDL-c/HDL-c ratio	1.29 (1.05, 1.60)	0.0159	1.22 (0.97, 1.54)	0.0932	1.25 (0.96, 1.65)	0.103
Non-HDL-c/HDL-c ratio						
0.85–2.03	1		1		1	
2.03–2.43	2.62 (1.35, 5.08)	0.0043	2.25 (1.14, 4.43)	0.0187	2.24 (1.13, 4.44)	0.0203
2.43–2.80	1.88 (0.94, 3.75)	0.0739	1.51 (0.74, 3.09)	0.2593	1.50 (0.73, 3.10)	0.269
2.80–3.26	2.84 (1.48, 5.47)	0.0017	2.47 (1.25, 4.85)	0.009	2.52 (1.26, 5.01)	0.0087
3.26–8.79	2.64 (1.37, 5.10)	0.0038	2.16 (1.09, 4.30)	0.0277	2.13 (1.04, 4.37)	0.0397

Data are Hazard ratio (95% CI), *P*-value, CI confidence interval

GBPs, gallbladder polyps; HDL-c, high-density lipoprotein -cholesterol

^a^Model adjusted for age and body mass index

^b^Model adjusted for age, body mass index, alanine aminotransferase, aspartate aminotransferase, gamma-glutamyl transpeptidase, alkaline phosphatase, triglyceride, uric acid, fasting plasma glucose, hepatitis B surface antigen, hypertension, and thickening of the gallbladder wall, smoking and drinking

**Table 4:** Multiple Cox regression analysis of non-HDL-c/HDL-c ratio and risk for presence of GBPs in subgroups

	No. of events	Hazard ratio (95% CI)	*P*-value	*P* for interaction
Age (yr)				0.8882
< 65	125	1.24 (0.93, 1.66)	0.1381	
> =65	12	1.32 (0.60, 2.88)	0.4889	
BMI (kg/m^2^)				0.3464
< 25	64	1.43 (0.97, 2.10)	0.0672	
> =25	61	1.10 (0.74, 1.63)	0.6333	
HBsAg				0.7683
0	124	1.27 (0.96, 1.69)	0.0998	
1	13	1.11 (0.48, 2.59)	0.802	
Hypertension				0.5645
0	107	1.20 (0.88, 1.64)	0.2442	
1	30	1.41 (0.87, 2.30)	0.1625	
TGW				1
0	137	1.25 (0.96, 1.65)	0.103	
1	0	– ^a^	1	
Smoking				0.6484
0	122	1.23 (0.93, 1.64)	0.1428	
1	14	1.49 (0.67, 3.31)	0.3232	
Drinking				0.8875
0	129	1.25 (0.95, 1.65)	0.1093	
1	7	1.37 (0.40, 4.68)	0.6176	

*P* for interaction stands for interaction between the non-HDL-c/HDL-c ratio and each subgroup

GBPs, gallbladder polyps; HDL-c, high-density lipoprotein -cholesterol; BMI, body mass index; HBsAg, hepatitis B surface antigen; TGW, thickening of the gallbladder wall

Data are adjusted for age, body mass index, alanine aminotransferase, aspartate aminotransferase, gamma-glutamyl transpeptidase, alkaline phosphatase, triglyceride, uric acid, fasting plasma glucose, hepatitis B surface antigen, hypertension, and thickening of the gallbladder wall, smoking and drinking

^a^The model failed because of the small sample size

## Results

### Characteristics of individuals by quintiles of the non-HDL-c/HDL-c ratio

Table 1 compares the baseline characteristics of individuals by quintiles of the non-HDL-c/HDL-c ratio. Significant differences were observed among the groups, except for the age, ALP, HBsAg, hypertension, TGW, smoking and drinking groups. Subjects in the higher quintiles were more likely to have higher BMI, ALT, AST, GGT, TG, TC, LDL-c, non-HDL-c, UA, and FPG values and lower HDL-c values than those in the lowest quintile (*P* < 0.001). Compared with that in the lowest quintile, the incidence of GBPs significantly increased in other quintiles (3.50% vs. 8.70, 6.38, 9.36, and 8.75% for quintile 1 vs. quintile 2, quintile 3, quintile 4, and quintile 5, respectively; *P* = 0.012).

### The prevalence of GBPs and the rise of the non-HDL-c/HDL-c ratio

Figure 1 presents the incidence of GBPs according to quintiles of the non-HDL-c/HDL-c ratio. The proportion of patients who had new-onset GBPs at the end of follow-up in quintile 2 (8.70%, *P* = 0.0260), quintile 4 (9.36%, *P* = 0.0095) and quintile 5 (8.75%, *P* = 0.0224) was significantly higher than that in quintile 1 (3.50%).

### Baseline data of subjects with or without GBPs after follow-up

The general characteristics of the subjects with or without GBPs are summarized in Table 2. Compared with subjects without GBPs, those who developed GBPs after follow-up had significantly higher TG levels and non-HDL-c/HDL-c ratios (*P* < 0.05).

### The prevalence of GBPs according to age groups

There was no difference in age between groups with or without GBPs. In Fig. 2, six groups were defined based on age. The prevalence of GBPs was 1.57, 8.33, 8.70, 8.57, 5.38 and 4.00% in the < 30 years group, 30–39 years group, 40–49 years group, 50–59 years group, 60–69 years group, and > 70 years group, respectively (*P* = 0.022). The prevalence of GBPs showed a linearity increment (*P* < 0.01) with age, got to peaks in the 30–39 years group, 40–49 years group and 50–59 years group, and then declined slightly.

### Unadjusted correlation between baseline data and GBPs

The results of a univariate analysis are shown by the forest plot in Fig. 3. The results showed that the non-HDL-c/HDL-c ratio (HR = 1.29, 95% CI, 1.05–1.60, *P* = 0.0159) was positively correlated with GBPs. This study also showed that age, BMI, ALT, AST, GGT, ALP, LDL-c, TC, HDL-c, UA, FPG, HBsAg, hypertension, TGW, smoking and drinking parameters were not associated with the risk of GBP formation, whereas the TG level (HR = 1.08, 95% CI, 1.00–1.17, *P* = 0.0510) and non-HDL-c level (HR = 1.22, 95% CI, 1.00–1.50, *P* = 0.0558) had marginally significant associations with the risk of GBPs.

### Independent relation between the non-HDL-c/HDL-c ratio and GBPs

The results of the multiple Cox proportional hazards models are shown in Table 3. With the lowest quintile as a reference, unadjusted Cox regression analysis showed that the HRs were 2.62 for quintile 2 (95% CI: 1.35–5.08, *P* = 0.0043), 1.88 for quintile 3 (95% CI: 0.94–3.75, *P* = 0.0739), 2.84 for quintile 4 (95% CI: 1.48–5.47, *P* = 0.0017) and 2.64 for quintile 5 (95% CI: 1.37–5.10, *P* = 0.0038). In the fully adjusted model, the HRs were 2.24 for quintile 2 (95% CI: 1.13–4.44, *P* = 0.0203), 1.50 for quintile 3 (95% CI: 0.73–3.10, *P* = 0.269), 2.52 for quintile 4 (95% CI: 1.26–5.01, *P* = 0.0087) and 2.13 for quintile 5 (95% CI: 1.04–4.37, *P* = 0.0397). However, multiple Cox regression analysis demonstrated that the non-HDL-c/HDL-c ratio as a continuous variable was not significantly related to GBPs in the fully adjusted model (HR = 1.25, 95% CI: 0.96–1.65, *P* = 0.103).

### Association between the non-HDL-c/HDL-c ratio and GBPs in subgroups

In Table 4, whether there was any interaction that might affect the relation between the non-HDL-c/HDL-c ratio and GBPs was examined. No interaction was found in the subgroups based on age, BMI, HBsAg, hypertension, TGW, smoking and drinking parameters (*P* > 0.05).

## Discussion

This retrospective study is the first to show that a higher non-HDL-c/HDL-c ratio is independently related to a higher risk of GBP formation in Chinese men. This association persisted after adjustment for potential confounders. The effect of the non-HDL-c/HDL-c ratio was further expanded.

Non-HDL contains multiple lipoproteins, such as low-density lipoprotein (LDL), very-low-density lipoprotein (VLDL), intermediate-density lipoprotein (IDL) and lipoprotein (a). Secondary targets for lipid-lowering therapy include non-HDL-c [27], and increasing evidence has shown that the non-HDL-c/HDL-c ratio is superior to evaluate lipid-related disease risk. An observational study demonstrated that the non-HDL-c/HDL-c ratio was superior to the non-HDL-c level in predicting the risk of coronary heart disease among diabetes patients [28]. The ratio was demonstrated to have higher predictive value than traditional lipoprotein levels in metabolic syndrome and insulin resistance [25]. The non-HDL-c/HDL-c ratio could reflect the condition of cholesterol transport to some extent, which may contain more informative lipid turbulence information than non-HDL-c or other lipoprotein levels. GBPs are a common disease entity, some of which have malignant tendency. Although the risk factors for GBP formation have been uncertain, dyslipidaemia is strongly associated with GBPs [3, 6, 21, 29]. Therefore, it is meaningful to investigate the relation between the non-HDL-c/HDL-c ratio and incident GBPs.

The study of Khairy et al. [30] showed that 85.1% of GBP patients had increased blood cholesterol levels. Several studies reported that plasma HDL-c levels in the GBP group were dramatically lower than those in the control group, whereas LDL-c levels were obviously raised [3, 6, 29]. The pathogenesis of GBPs is complicated and involves many factors. The main type of GBP is cholesterol polyps, which are connected with the metabolism of cholesterol in bile. One of the characteristics of GBPs is mucosal hyperplasia, which accumulates excessive cholesterol esters in epithelial macrophages [31]. Excessive cholesterol is engulfed by macrophages and deposited in the gallbladder mucosa, which promotes hyperplasia of the gallbladder mucosa and damages the contractility of the gallbladder. In addition, the stasis of venous and lymphatic systems further destroys cholesterol absorption and secretion of the gallbladder mucosa, and GBPs occur [32]. The development of GBPs is believed to be associated with cholesterol from the bile or blood [30]. Cholesterolosis may result from the direct deposition of cholesterol from the plasma, similar to the formation of plaque in atherosclerosis [33]. Changes in liver cholesterol metabolism and mucosal esterification of free sterols in bile may induce occurrence and development in GBPs [34]. However, whether the cholesterol deposited in the gallbladder comes from the plasma or from the bile remains unclear. Most studies have paid more attention to the absorption and excretion of cholesterol by the gallbladder mucosa [35].

Previous research has demonstrated that increased TG level is associated with GBPs [21]. This study also showed that the TG level in the GBP group was much higher than that in the non-GBP group. The role of TG in GBP formation is unclear. There is a complex relationship between TG and insulin resistance. Enhanced TG mobilization may help improve insulin resistance [36]. TG might share a similar pathogenesis with insulin resistance. Many studies have reported that middle age is closely correlated with the occurrence of GBPs [12, 20, 21]. The present study also found evidence for this phenomenon. Along with the increase in age, the incidence of GBPs showed a downward trend after rising to a peak, which occurred between 30 and 59 years. This finding may be related to high work pressure, irregular lifestyle, hormone levels and immunity in a period of change. However, univariate analysis failed to find that age was a risk factor for GBP formation in the current study.

### Study strengths and limitations

This study has certain potential clinical implications. Exploring the influence of the non-HDL-c/HDL-c ratio on GBPs is meaningful for further research on the pathology of GBPs. Moreover, this research also provides guidance for the treatment of GBPs. Serum cholesterol levels are easily measured and can be used as an objective indicator. In addition, the non-HDL-c/HDL-c ratio can serve as a reference biomarker for GBPs during follow-up.

Several limitations for this study should be mentioned. First, as this was a single-centre cohort study in China, the results might not be directly applicable to other regions and ethnicities. Additional cohort studies of ethnically diverse adults in different regions are needed to confirm the generalizability of the study results. Second, the data for the study did not include waist circumference, blood pressure, insulin resistance, other drug use or possible familial predisposition. This study used hypertriglyceridemia, and low HDL-c and high FPG levels to define metabolic syndrome. Although metabolic syndrome is a risk factor for GBP formation [17], variables involved in the metabolic syndrome defined in this study were included in the statistical analysis. Third, GBPs were not diagnosed by biopsy, which may have led to misdiagnosis. Finally, unmeasured confounding factors might not have been fully addressed.

## Conclusions

In conclusion, according to the above analysis, these findings were the first evidence that a higher non-HDL-c/HDL-c ratio was connected to the risk of GBP formation among men in China. Prospective studies would be meaningful to confirm the actual risk of these patients and the effect of the non-HDL-c/HDL-c ratio. The present study was performed in male patients in China. It should be interesting to prove whether the same conclusion could be arrived for people in different regions, races and social classes.

## Data Availability

The data of the current study can be obtained from the corresponding author on reasonable request.

## Abbreviations

GBP: Gallbladder polyp
non-HDL-c/HDL-c ratio: non-high-density lipoprotein-cholesterol-to-high-density lipoprotein-cholesterol ratio
TG: Triglyceride
HR: Hazard ratio
CI: Confidence interval
HBsAg: Hepatitis B surface antigen
non-HDL-c: non-high-density lipoprotein-cholesterol
TC: Total cholesterol
HDL-c: High-density lipoprotein-cholesterol
BMI: Body mass index
LDL-c: Low-density lipoprotein-cholesterol
AST: Aspartate aminotransferase
ALT: Alanine aminotransferase
ALP: Alkaline phosphatase
GGT: Gamma-glutamyl transpeptidase
UA: Uric acid
FPG: Fasting plasma glucose
TGW: Thickening of the gallbladder wall
LDL: Low-density lipoprotein
VLDL: Very-low-density lipoprotein
IDL: Intermediate-density lipoprotein
